# Spontaneous State Detection Using Time-Frequency and Time-Domain Features Extracted From Stereo-Electroencephalography Traces

**DOI:** 10.3389/fnins.2022.818214

**Published:** 2022-03-17

**Authors:** Huanpeng Ye, Zhen Fan, Guangye Li, Zehan Wu, Jie Hu, Xinjun Sheng, Liang Chen, Xiangyang Zhu

**Affiliations:** ^1^State Key Laboratory of Mechanical System and Vibration, School of Mechanical Engineering, Shanghai Jiao Tong University, Shanghai, China; ^2^Department of Neurosurgery of Huashan Hospital, Fudan University, Shanghai, China

**Keywords:** stereo-electroencephalography, brain-computer interface, feature evaluation, time-domain feature, high-gamma

## Abstract

As a minimally invasive recording technique, stereo-electroencephalography (SEEG) measures intracranial signals directly by inserting depth electrodes shafts into the human brain, and thus can capture neural activities in both cortical layers and subcortical structures. Despite gradually increasing SEEG-based brain-computer interface (BCI) studies, the features utilized were usually confined to the amplitude of the event-related potential (ERP) or band power, and the decoding capabilities of other time-frequency and time-domain features have not been demonstrated for SEEG recordings yet. In this study, we aimed to verify the validity of time-domain and time-frequency features of SEEG, where classification performances served as evaluating indicators. To do this, using SEEG signals under intermittent auditory stimuli, we extracted features including the average amplitude, root mean square, slope of linear regression, and line-length from the ERP trace and three traces of band power activities (high-gamma, beta, and alpha). These features were used to detect the active state (including activations to two types of names) against the idle state. Results suggested that valid time-domain and time-frequency features distributed across multiple regions, including the temporal lobe, parietal lobe, and deeper structures such as the insula. Among all feature types, the average amplitude, root mean square, and line-length extracted from high-gamma (60–140 Hz) power and the line-length extracted from ERP were the most informative. Using a hidden Markov model (HMM), we could precisely detect the onset and the end of the active state with a sensitivity of 95.7 ± 1.3% and a precision of 91.7 ± 1.6%. The valid features derived from high-gamma power and ERP in this work provided new insights into the feature selection procedure for further SEEG-based BCI applications.

## 1. Introduction

Brain-computer interfaces (BCIs) aim to build a bridge between the human brain and the external world by directly interpreting neural signals from the brain. Over the past two decades, intracranial electroencephalography (iEEG) has been proved to be a reliable brain signal acquisition technique for BCI (Schalk and Leuthardt, 2011; Herff et al., 2020). Compared with scalp EEG, iEEG has millimeter-level spatial resolution (Parvizi and Kastner, 2018), a higher signal-to-noise ratio, and a broader frequency range (up to 500 Hz) (Urrestarazu et al., 2007). Typically, iEEG recordings are categorized into electrocorticography (ECoG) and stereo-electroencephalography (SEEG). Due to sufficient information from low-frequency oscillations and broadband gamma activities (Parvizi and Kastner, 2018), decoding studies using iEEG have achieved remarkable performances in motor (Chestek et al., 2013; Branco et al., 2017), visual perception (Miller et al., 2016), intonational speech prosody (Tang et al., 2017), and mood paradigms (Sani et al., 2018). ECoG electrode arrays are surgically placed above or below the dura matter to record neural activities of the cortex, and hence can not access the neural activities of subcortical structures. In comparison, as a minimally invasive approach with less infection and hemorrhage risk (Arya et al., 2013; Mullin et al., 2016), SEEG measures neural activities directly by inserting depth electrodes containing multiple recording contacts into the human brain. Therefore, SEEG can simultaneously measure the neural information from both cortical and deeper structures, including the insula and hippocampus. Especially, SEEG is suitable for simultaneous recording of two brain hemispheres (Nair et al., 2008; Minotti et al., 2018). With the increasing use of SEEG in monitoring medication-resistant epilepsy, it becomes possible to evaluate the potential of SEEG signals in BCI applications.

Several SEEG-based BCI studies have been reported. Within these studies, band power features and event-related potentials (ERP) features are the most commonly used features. In typical motor BCI paradigms, two-dimensional cursor trajectory (Vadera et al., 2013), different hand gestures (Li et al., 2017b; Wang et al., 2020), and imagined grip force level (Fischer et al., 2017) have been successfully decoded using the power of classic motor-related brain oscillations such as alpha (8–12 Hz) and beta (13–30 Hz) power, as well as high-gamma (60–200 Hz typically) power. Besides these band power features, ERP recorded in the sensorimotor cortex has also been used to decode executed and imagined grip forces (Murphy et al., 2016). Moreover, significant ERPs obtained in the middle temporal region (Li et al., 2017a), the ventricle (Shih and Krusienski, 2012), and the hippocampus (Krusienski and Shih, 2011) have been adopted in the building of visual spellers.

Notably, the above SEEG-based BCI studies only take the average amplitude of the band powers or ERPs as features. While other summative measurements of the band power and ERP traces in a time window have been successfully used in BCI studies based on either EEG or ECoG and exhibited remarkable decoding performance. For example, the root mean square (RMS) of ERP can provide informative features in visual speller and movement tasks (Mak et al., 2012; Bascil, 2018). The slope between peak and valley points and the slope from linear regression of EEG in a time window also play a role in decoding (Phillips et al., 2012; Adam et al., 2014). Moreover, the line-lengths of ECoG signals have been used as features in hand motion decoding (Xie et al., 2015). To date, it is largely unknown whether or not the above time-domain features could provide selective information for SEEG-based BCI. Additionally, when these time-domain features are extracted from traditionally used band power traces, novel time-frequency features (e.g., line-length of high-gamma power trace) can be generated. Also, it is still unknown whether such time-frequency features can play a role in SEEG-based BCI applications.

Therefore, this study aimed to evaluate the validity of various features of SEEG recordings, including time-domain features extracted from ERP trace and time-frequency features extracted from band power traces (high-gamma, beta, and alpha). To this end, different features were extracted from continuous SEEG signals, and we classified the active state (including activations to two types of auditory name stimuli) and idle state from continuous SEEG data, and the evaluation indicators were set as the sensitivity and precision for active state detection. Moreover, to find optimal decoding settings, performances of different window lengths and classifiers were compared as well. The results showed that the onset and the end of the active state could be precisely detected using a hidden Markov model (HMM). By splitting the active state into two different states, a three-class (own name, other name, and idle) classification still demonstrated the effectiveness of the proposed feature combination and classification scheme. This study validated the spontaneous decoding of subjects' active state and idle state under auditory stimuli using SEEG recordings, and systematically evaluated various features under the same paradigm.

## 2. Materials and Methods

### 2.1. Subjects

**Figure 1:**
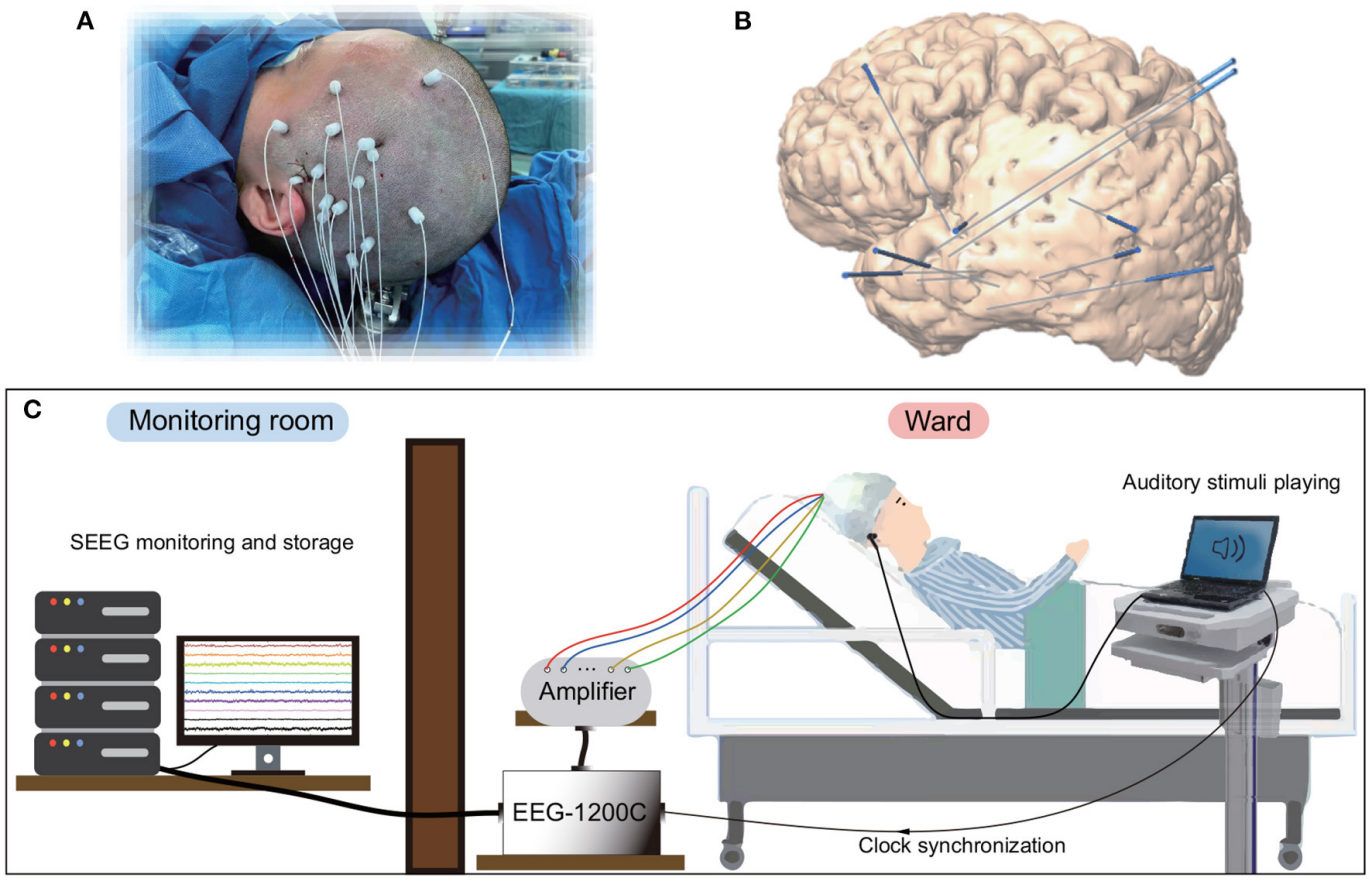
Electrode implantation and recording environments for SEEG. **(A)** Surgical implantation of electrode shafts. **(B)** A sample of 3D brain model with implanted electrode shafts. **(C)** A patient and the whole recording system in a clinical environment.

Seven right-handed subjects (Table 1) participated in this study. All subjects were intractable epilepsy patients undergoing SEEG monitoring for seizure localization. SEEG electrodes implanting configurations were determined strictly for diagnostic purposes rather than the needs of this study. Figure 1 shows an example of SEEG electrodes placement and recording environments. All subjects signed informed consent, which was approved by the Ethics Committee of Huashan Hospital, Shanghai, China (No. KY2019518).

**Table 1:** Subject demographics, implanting information, and neural recording details.

**Subject**	**Age**	**Implanting**	**Sampling**	**Trial**	**Cue and**	**Stimulus**	**Inter-trial**
	**(years)**	**hemisphere**	**frequency**	**size**	**preparation**	**duration (ms)**	**interval (ms)**
S1	31	Left (144)	2,000	60	−	2,000	2,000
S2	30	Left (138), Right (32)	2,000	80	−	2,000	2,000
S3	24	Right (104)	2,000	120	−	1,000	1,000
S4	24	Right (108)	1,000	120	−	1,000	1,000
S5	33	Left (150)	2,000	120	*	1,000	1,100−1,300
S6	24	Left (90)	2,000	120	*	1,000	1,100−1,300
S7	32	Left (30), Right (96)	2,000	120	*	1000	1,100−1,300

The numbers in brackets in the “Implanting hemisphere” column indicate the number of contacts in the hemisphere. The “*” or “−” in the “Cue and preparation” column indicates the existence or non-existence of the cue and preparation time before the name presentation, respectively.

### 2.2. Experimental Paradigm

**Figure 2:**
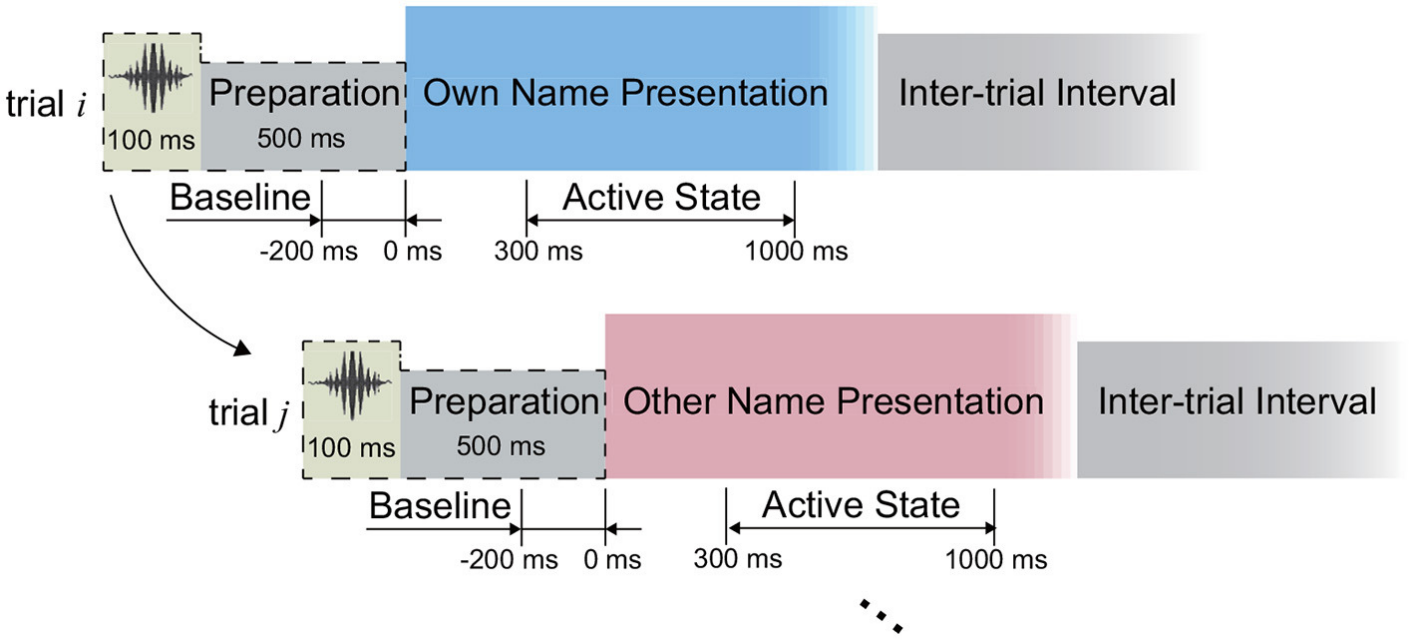
Experimental paradigm. The periods from the 300 to 1,000 ms following the stimuli onsets were defined as active states during the signal processing.

In this study, we implemented an acoustic name presentation experiment (Figure 2). More specifically, during the experiment, all subjects received two kinds of auditory stimuli, where one was the subject's own name, and the other was a stranger's name with the same length as the own name. The name stimulus was presented to each subject by an in-ear headphone. In each trial, the subject received the stimulus first and then could relax in the inter-trial interval (Figure 2). The two names were repeated for equal trials in a pseudo-random sequence. The trial size, duration of the stimulus, and inter-trial interval of each subject were shown in Table 1. After preliminary analysis for the first two subjects S1 and S2, we found that 1-s stimulus duration was enough to elicit the subject's complete response, and 1-s inter-trial interval was enough for the response to revert to the baseline. Therefore, the stimulus duration and inter-trial interval were set as 1 s for the following subjects. Furthermore, to avoid the subject's adaption, another floating inter-trial interval from 100 to 300 ms was added (Nowicka et al., 2016) for subjects S5-S7, and these subjects received a 100-ms cue (a short burst of sound) and 500-ms preparation time before the name presentation to attract attention.

### 2.3. Data Recording and Electrode Localization

SEEG data were recorded with a clinical recording system (EEG-1200C, Nihon Kohden, Irvine, CA). The cut-off frequency of the hardware filter of the recording system was 0–3,000 Hz, and SEEG data were digitized at 1,000 or 2,000 Hz. Each depth electrode shaft contains 8–16 contacts. Each contact is 2 mm long with a 0.8 mm diameter and 1.5 mm spacing distance. For signal recording, the ground electrode was placed at the location of Fpz on the scalp; SEEG signals were referenced against the average of two white matter contacts that were adjacent to each other and located remotely from the suspected epileptogenic foci and gray matter. This referencing technique was the same for all channels and is commonly used by the surgeons at Huashan hospital. For each subject, using pre-implant MRI and post-implant CT images, we first rebuilt the individual brain by performing brain reconstruction and segmentation in Freesurfer (Fischl et al., 2002), and then identified the 3D coordinates and the anatomical labels within the brain for all SEEG contacts using iEEGview Matlab toolbox (Li et al., 2019).

### 2.4. Pre-processing and Channel Selection

We first investigated the capability of SEEG to distinguish the active state and the idle state, where the periods from 300 to 1,000 ms following the name stimuli onsets were used for the active state calculation consistently (Figure 2). To do this, for each type of signal trace, including the high-gamma (60–140 Hz), beta (13–30 Hz), and alpha (8–12 Hz) band power, as well as the ERP, we selected the corresponding active channels separately, where the channels presenting significantly different amplitudes of signal traces between the active period and the idle period were identified. In detail, take the high-gamma power as an example to show the channel selection. In the first step of channel selection, for each channel, the amplitude of high-gamma power was extracted as follows: (1) the signals were filtered by a 50 Hz comb notch filter to remove the possible line noises and their harmonics; (2) we re-referenced the signals using the Laplacian reference method (Li et al., 2018); (3) we band-pass filtered the signals between 60 and 140 Hz using a 6th-order Butterworth filter and thus high-gamma signals were generated. Then we applied the Hilbert transform on the high-gamma signal of each channel to extract its absolute amplitude. By squaring the absolute amplitude, we obtained the high-gamma power trace of each channel. Regarding the ERP trace, raw voltages of the active channels were subjected to a 50-Hz comb notch filter and a high-pass filter (0.5 Hz), and then a Laplacian re-referencing scheme, and thus the ERP traces were generated. Then for each type of signal trace, we divided the trace into trials according to the markers at the stimulus onset. For each trial, the 200-ms period preceding the stimulus onset was considered as the baseline, namely the idle state period (Figure 2). We normalized the trace using Z-scored transformation against its baseline. Finally, the trace of each trial was convoluted with an 80-ms Gaussian window for smoothing (Miller et al., 2016).

**Figure 3:**
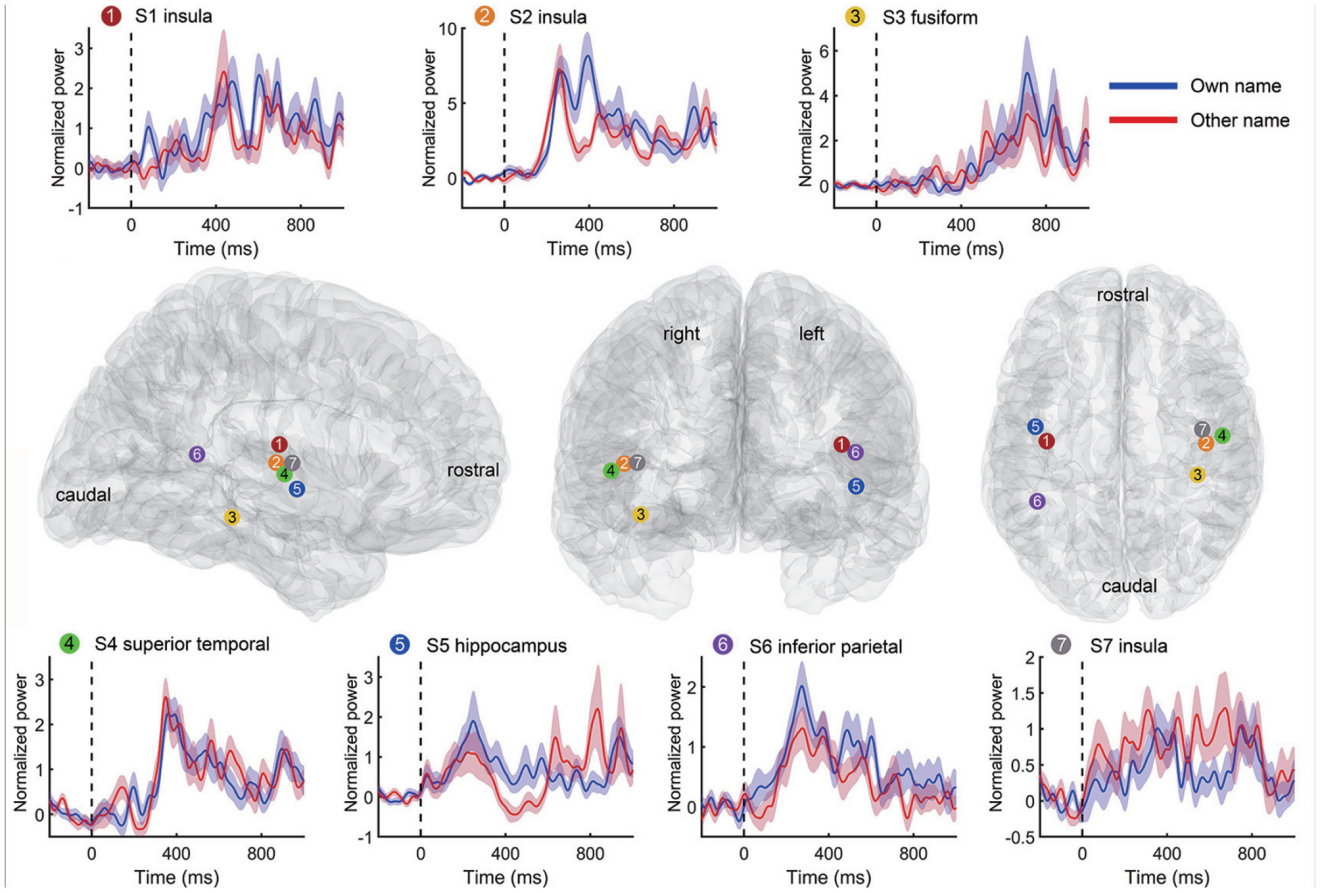
Representative high-gamma responses and positions of active channels for each subject. Each time-power plot in the top and bottom rows reflects a subject's high-gamma power (mean and standard error) across trials from an active channel. The responses to own name and other's name stimuli were displayed separately. The black vertical line indicates the stimuli onset. The standard Montreal Neurological Institute (MNI) brain models in the middle row show positions of the above channels in the sagittal, coronal, and transverse view. Channel positions of different subjects are marked with different colors and numbers.

In the second step of channel selection, a random permutation test was used to check whether the single channel was active indeed by using all trials within the channel repeatedly (Schalk et al., 2007; de Pesters et al., 2016; Li et al., 2018). Therefore, we could evaluate each channel independently by this permutation test. Take the high-gamma power as an example. For each channel, the average high-gamma power amplitude of the active state period and the idle state period in each trial were set as *x* and *y*, respectively. We first concatenated all *x* and *y* of all trials as *z*, and then correlated *z* with the corresponding labels to obtain the observed Spearman *r*-value. Second, the active/idle labels (e.g., −1/1 for active/idle state respectively) were randomly shuffled, and the *r*-value between *z* and randomized labels was calculated. Then we repeated this randomization step 1,000 times, thus generating a Gaussian distribution with 1,000 surrogate *r*-values. The observed *r* was considered statistically significant if it belonged to the 95th percentile of the Gaussian distribution (*p* < 0.05 after Bonferroni correction), and correspondingly, these channels were considered active and selected for further analysis. The high-gamma responses of a representative active channel from different subjects are shown in Figure 3. To avoid too much redundant information, we only kept the ten most informative channels with the smallest *p*-values for each type of signal trace if the number of active channels was larger than ten. Only the kept active channels were used in the following feature extraction. Regions containing active channels are shown in Table 2.

**Table 2:** Regions containing active channels used for classification.

**Subject**	**Regions containing active channels**	**Subject**	**Regions containing active channels**
S1	Left insula Left putamen Left bankssts Left superior temporal Left supramarginal Left pars opercularis	S2	Left insula Left supramarginal Left pars opercularis Left superior temporal Left transverse temporal Right insula Right supramarginal
S3	Right insula Right fusiform Right superior temporal Right inferior parietal Right inferior temporal Right parahippocampal Right transverse temporal	S4	Right fusiform Right inferior parietal Right inferior temporal Right superior temporal Right superior parietal Right transverse temporal
S5	Left insula Left hippocampus Left superior temporal Left transverse temporal	S6	Left bankssts Left inferior parietal Left middle temporal
S7	Right superior temporal Right insula Right supramarginal		

*The “bankssts” of subject S1and S6 is the abbreviation for banks of the superior temporal sulcus. As long as a channel was decided as active using any one of the four signal traces, the corresponding anatomical label was shown in this table*.

### 2.5. Feature Extraction

**Figure 4:**
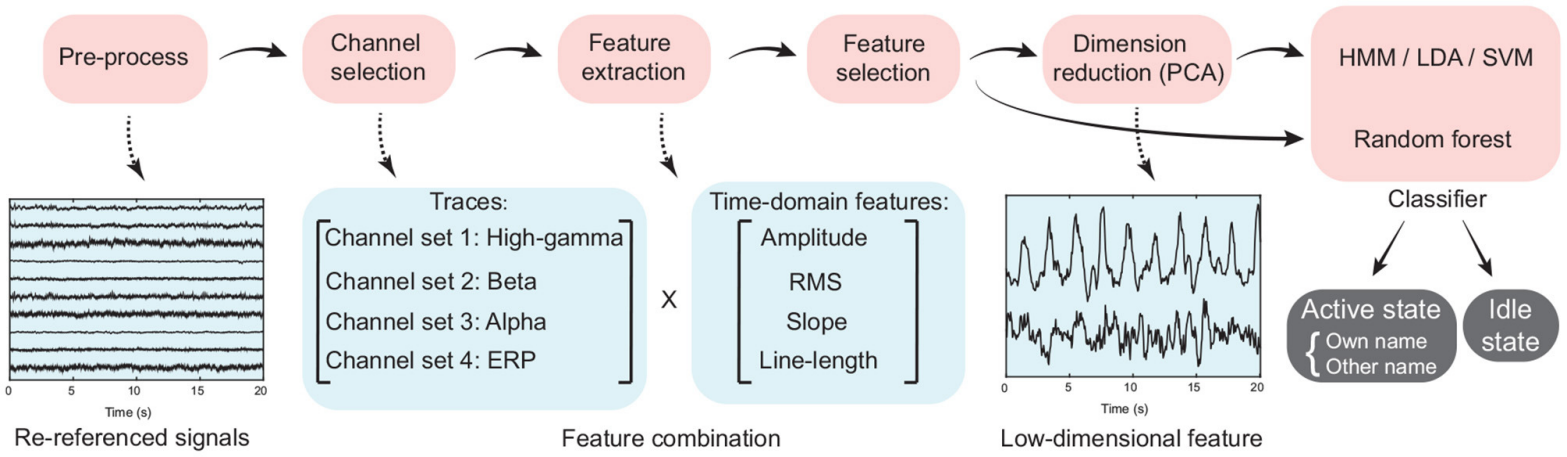
The flow chart of signal processing and classification procedure. Time-domain features, including the average amplitude, root mean square, slope of linear regression, and line-length were extracted from the high-gamma, beta, and alpha band power traces, as well as the event-related potential.

Using the selected active channels for high gamma, beta, and alpha bands, as well as ERP activity separately, we extracted the features input into the classifiers, where the idle state periods were treated as the periods except the active state periods (Figure 2). In detail, ERP and power traces of high-gamma, beta, and alpha bands in a time window were extracted from the pre-processed signals using the corresponding active channel set respectively (Figure 4, Section 2.4). Then four time-domain features, including the average amplitude, RMS, slope, and line-length were extracted from each type of signal trace (high-gamma power, beta power, alpha power, and ERP). Therefore, for each type of signal trace, an active channel generated four features (Figure 4), and the feature combination had 16 types of time-frequency and time-domain features in total. For each feature dimension, according to its values and active/idle labels (−1/1) across trials in the training set, here we implemented a random permutation test again and thus generated the *p*-value for the feature dimension. Only ten features with the smallest *p*-values were kept. Finally, for convenience of visualization, a principal component analysis (PCA) was used for dimension reduction, and principal components that corresponded to more than 95% of explained variance were used for classification.

### 2.6. Classification and Evaluation

Three-fold cross-validation was used during the classification procedure (Miller et al., 2016). More specifically, the continuous data stream during the whole experiment was divided into three consecutive streams evenly, where two streams were used to train the classifier, and the remaining one was used as the testing set. In this way, there was no overlap between sliding windows of the training set and test set. To comprehensively evaluate the decoding capability of obtained SEEG signals, we compared the influence of different windows lengths on the classification performance. In detail, window lengths from 150 to 500 ms were used to generate the samples used for training and testing, where the overlap time of the sliding window was 50 ms. Moreover, to find which kind of classifiers was more proper for decoding in the current task, under each window length, we also compared the performance of a series of classifiers, including HMM, linear discriminant analysis (LDA), support vector machine (SVM), and random forest (RF).

Details on the fundamentals and implementation of HMM are available in Rabiner (1989). In brief, the probability of the model's current state is obtained by multiplying the transition probability of the previous state to the current state and the probability of detecting the current features given the current state. In our cases, to initialize the HMM based on continuous variables, the matrix of state transition probability and the probability density functions of features under different states were required (Rabiner, 1989). Therefore, we first estimated the probability density functions of features under the two states separately by the Gaussian mixture model (GMM), a parametric probability density function represented by a weighted sum of Gaussian component densities (Reynolds, 2009). And then, the matrix of state transition probability was generated by computing state transition times according to active/idle labels of the training set.

To evaluate the detection performance for the active state, the sensitivity and precision of the classification were calculated, where the metrics are defined as follows:


(1) $$\begin{array}{rcl} Sensitivity & = & \frac{\text{TP}}{\text{TP}+\text{FN}}\times 100\% \end{array}$$

(2) $$\begin{array}{rcl} Precision & = & \frac{\text{TP}}{\text{TP}+\text{FP}}\times 100\% \end{array}$$

**Figure 5:**
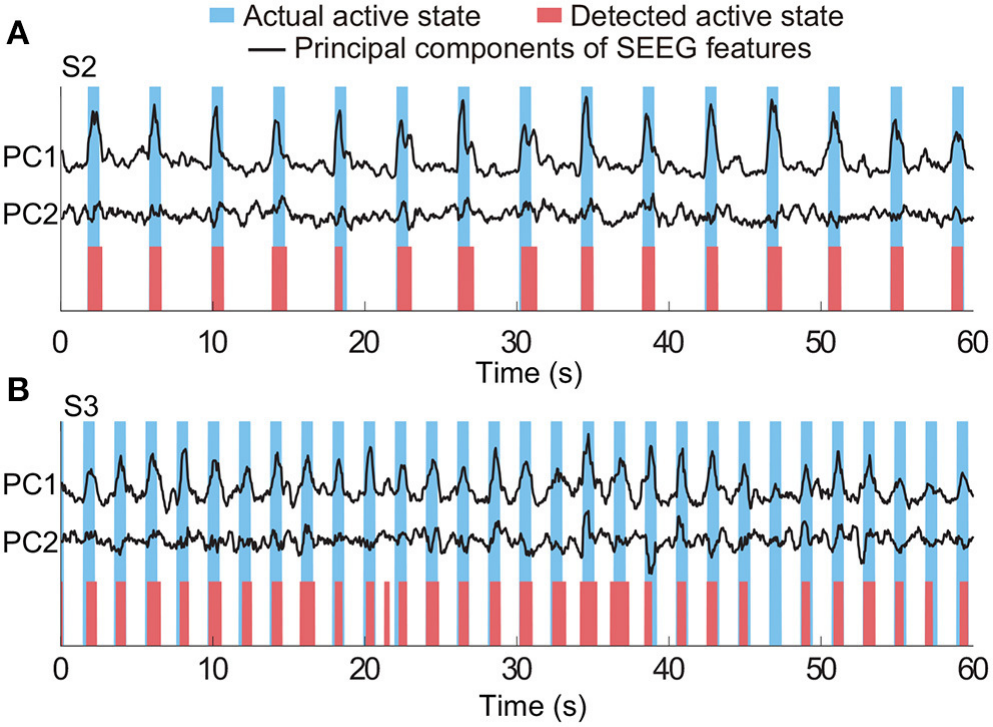
Representative results of state detection. Light blue and red bars respectively indicate the actual active state and detected active state by the HMM classifier. Periods with white indicate the actual and detected idle states. Panels **(A,B)** are the results from two typical subjects (S2 and S3). There is no FN and FP detection in **(A)**, and there is one FN detection and one FP detection in **(B)**.

In the equations, true positives (TP) was the number of active state periods identified by both algorithm and actual labels as well; false negatives (FN) was the number of actual active state periods, which were missed by the algorithm; the false positive (FP) was the number of actual idle state periods, which were identified as active state periods by the algorithm. Representative detection results of subjects S2 and S3 are shown in Figure 5. In the examples, all active state periods of S2 are TP detections, and S3 has one FP detection and one FN detection.

Besides the sensitivity and precision, we also calculated the accuracy indicator. The total accuracy for all periods of active and idle states was defined as follows:


(3) $$\begin{array}{r} Accuracy=\frac{\text{TP}+\text{TN}}{\text{TP}+\text{FP}+\text{TN}+\text{FN}}\times 100\% \end{array}$$

True negatives (TN) was the number of idle state periods identified by both algorithm and actual labels as well. Using TP, FN, FP, and TN, we also calculated the confusion matrix to show the classification accuracy of each type of state.

Additionally, we have calculated the chance levels of the sensitivity, precision, and total accuracy indicators by permutation tests. In detail, within each subject, we randomly shuffled the actual labels (active or idle) of all sliding windows before the feature extraction and classification. Then we re-implemented the feature extraction and classification. The evaluation indicators were calculated with actual labels and the outputs of the classifier. This procedure was repeated 1,000 times, and the significance level of evaluation indicators corresponded to the 95th percentile (*p* < 0.05) of the empirical distribution established by randomly permuting the labels (Combrisson and Jerbi, 2015; Branco et al., 2016).

Further, using the identified window length and classifier, we calculated the time difference between the detected active state onset and the actual active state onset (Onset_*detected*_ minus Onset_*actual*_), as well as the time difference between the detected active state end and the actual active state end (End_*detected*_ minus End_*actual*_). We only calculated the time differences when the actual active state and the detected active state had overlapping. Additionally, when the actual active state and the detected active state had an onset difference or end difference larger than 400 ms, the averaged time difference would be expanded, in the meantime, an extra FP or FN detection would be counted to make the evaluation stricter. In a few cases, one period of detected active state could penetrate two periods of actual active state. In such cases, only 1 TP detection and 1 FP detection were counted, and the time difference of the onset point was equal to the onset of the detected active state minus the onset of the first period of actual active state, and the time difference of the end point was equal to the end of the detected active state minus the end of the second period of actual active state.

### 2.7. Comparison With Traditional Frequency-Domain and Time-Frequency Features

Frequency-domain features have been widely used in decoding the field potentials. Also, both short-time Fourier transform (STFT) and wavelet transform have been widely used for extracting time-frequency domain features in field potentials (EEG, ECoG, SEEG, and local field potential) analysis. Therefore, we compared the combination of time-domain and time-frequency features of this study with other frequency-domain and time-frequency features. In sliding windows, spectral amplitudes in the frequency domain were extracted by autoregressive (AR) model, and time-frequency features were extracted by STFT and wavelet transform separately. Details of implementation of the three approaches are as follows.

*AR model:* for each time window, we converted the re-referenced SEEG data into the frequency domain with an AR model of order 100 (Schalk et al., 2007). Using the AR model (built by the Matlab function *pyulear*), we calculated the spectral amplitudes in the interested frequency bands (high-gamma, beta, and alpha) with 1 Hz bins. In each frequency band, we averaged the spectral amplitudes across frequency bins. *STFT:* we used the Matlab function *spectrogram* to implement the STFT in each time window. In detail, the 400-ms re-referenced SEEG data was divided into sections (length = number of data points/4.5, referring to the example of function introduction), and the sections were windowed using a Hamming window. We specified 50% overlap between contiguous sections. Thus the time-variant power spectral densities (PSD) were extracted from the high-gamma, beta, and alpha bands, respectively. In each frequency band, we averaged the PSD across frequency bins and time points to generate the feature for the 400-ms window. *Wavelet transform:* the Matlab function *cwt* was used to implement the continuous wavelet transform in each time window. The *cwt* was obtained using the analytic Morse wavelet with the symmetry parameter equal to 3 and the time-bandwidth product equal to 60. The wavelet coefficients in the high-gamma, beta, and alpha bands were extracted separately. After that, in each frequency band, we averaged the absolute value of wavelet coefficients across frequency bins and time points.

For each frequency band, only features of its own active channels (Section 2.4, Figure 4) were kept. After extracting frequency-domain or time-frequency features across all sliding windows by each approach, we then re-executed the same PCA dimension reduction and GMM-HMM classification scheme for each frequency band separately.

### 2.8. Detection Involving Different Active States

To further demonstrate the effectiveness of the proposed feature combination and GMM-HMM classification scheme, we added a three-class classification, where the active state was split into the own name state and the other's name state. We termed different states as idle state, own name state, and other name state, respectively. Before the detection, the same feature extraction process described above was used (Section 2.5, Figure 4). But slightly differently, the feature selection process was extended for the current classification scheme on the basis of the described method (Section 2.5), where the permutation test for feature selection was conducted three times at each feature dimension here. In detail, using permutation tests, the *p*-values (Bonferroni corrected) of comparisons between idle/own name states were first computed at all feature dimensions separately, and ten feature dimensions with the smallest *p*-values were selected. In the same way, we also computed *p*-values for comparisons between idle/other name states and own/other name states to select ten feature dimensions respectively. As the above classification scheme, only one HMM classifier was used, which had three types of outputs (own name, other name, and idle state).

## 3. Results

### 3.1. Influence of Window Lengths and Classifiers on Detection Performance

**Figure 6:**
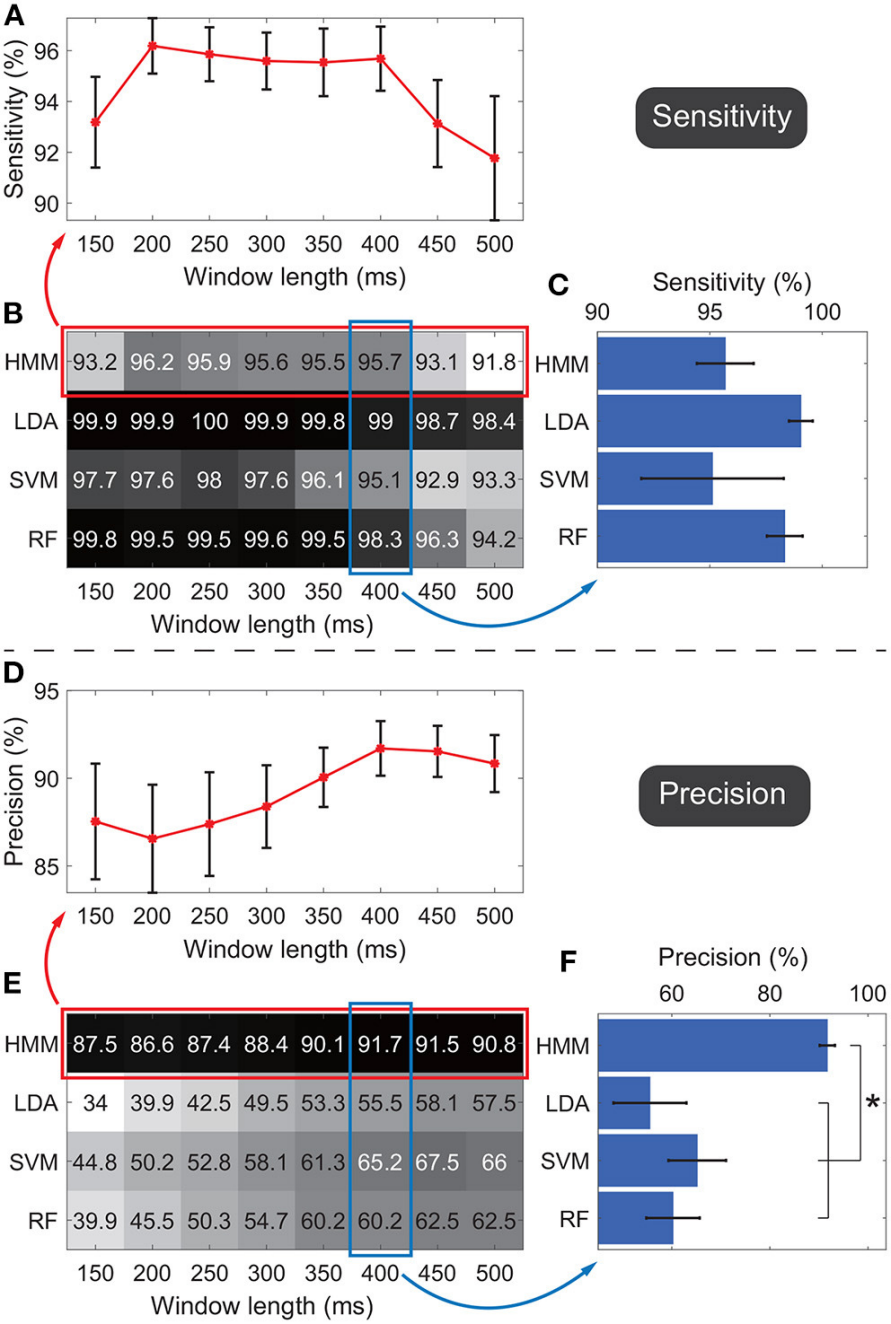
Sensitivities and precisions under different window lengths and different classifiers. Panels **(A–C)** indicate the evaluations of sensitivities. Panels **(D–F)** indicate the evaluations of precisions. Panels **(B,E)** show the average sensitivity and precision across subjects respectively, where the rows in the matrix indicate results of the four classifiers and the columns indicate the results under different window lengths. Darker color indicates a higher value. Panels **(A,D)** further visualize performances of HMM with standard errors across subjects, where all window lengths were used. Panels **(C,F)** further visualize performances of four classifiers with standard errors across subjects, where only the 400-ms window was adopted. *p < 0.05.

Figure 6 shows the detection performances of the active state against the idle state. Figures 6B,E show the average sensitivity and precision across subjects respectively, where the rows indicate results of the four classifiers and the columns indicate the results under different window lengths. In general, results suggested that the sensitivity and precision did not simply follow an upward or downward tendency as the window length changed for the same classifier. In general, for different classifiers, we observed the HMM outperformed all the other classifiers. Thus we focused on the performance of HMM. To show the standard error across subjects, the average sensitivities and precisions of HMM under all window length settings were shown in the line charts (Figures 6A,D). Under each window length, the sensitivity and precision of HMM were high enough (around 90%), and the sum of sensitivity and precision reached its maximum under the 400-ms window, where the sensitivity was 95.7 ± 1.3% and the precision was 91.7 ± 1.6% (mean ± standard error). Thus only this window length was adopted in the following classification.

The performance of HMM and other classifiers was further evaluated under the 400-ms window. The four classifiers' sensitivities and precisions were shown in bar charts to display the standard error across subjects (Figures 6C,F). The average sensitivity of HMM (95.7 ± 1.3%, ) was lower than ones of LDA (99 ± 0.5%) and RF (98.3 ± 0.8%), and higher than the one of SVM (95.1 ± 3.2%). The four groups of sensitivities met the homogeneity of variances (*p* = 0.06, Levene test), and thus one-way ANOVA suggested that there was no significant difference among sensitivities of the four classifiers (*F* = 1.2, *p* = 0.33).

Regarding the precision, HMM resulted in the highest performance (91.7 ± 1.6%, Figure 6F). The four groups of precisions did not meet the homogeneity of variances (*p* = 0.02, Levene test), and thus non-parametric tests (Mann–Whitney *U*-test) suggested that *p*-values for comparisons between HMM against LDA, SVM, and RF were 0.003, 0.027, and 0.008 respectively. SVM resulted in the second-highest average precision, whereas the value was only 65.2%, much lower than the value of HMM. Taken together, the results demonstrated that, among all the classifiers, HMM could achieve the highest precision and high enough sensitivity. Therefore, HMM was adopted as the classifier in the following calculation.

**Figure 7:**
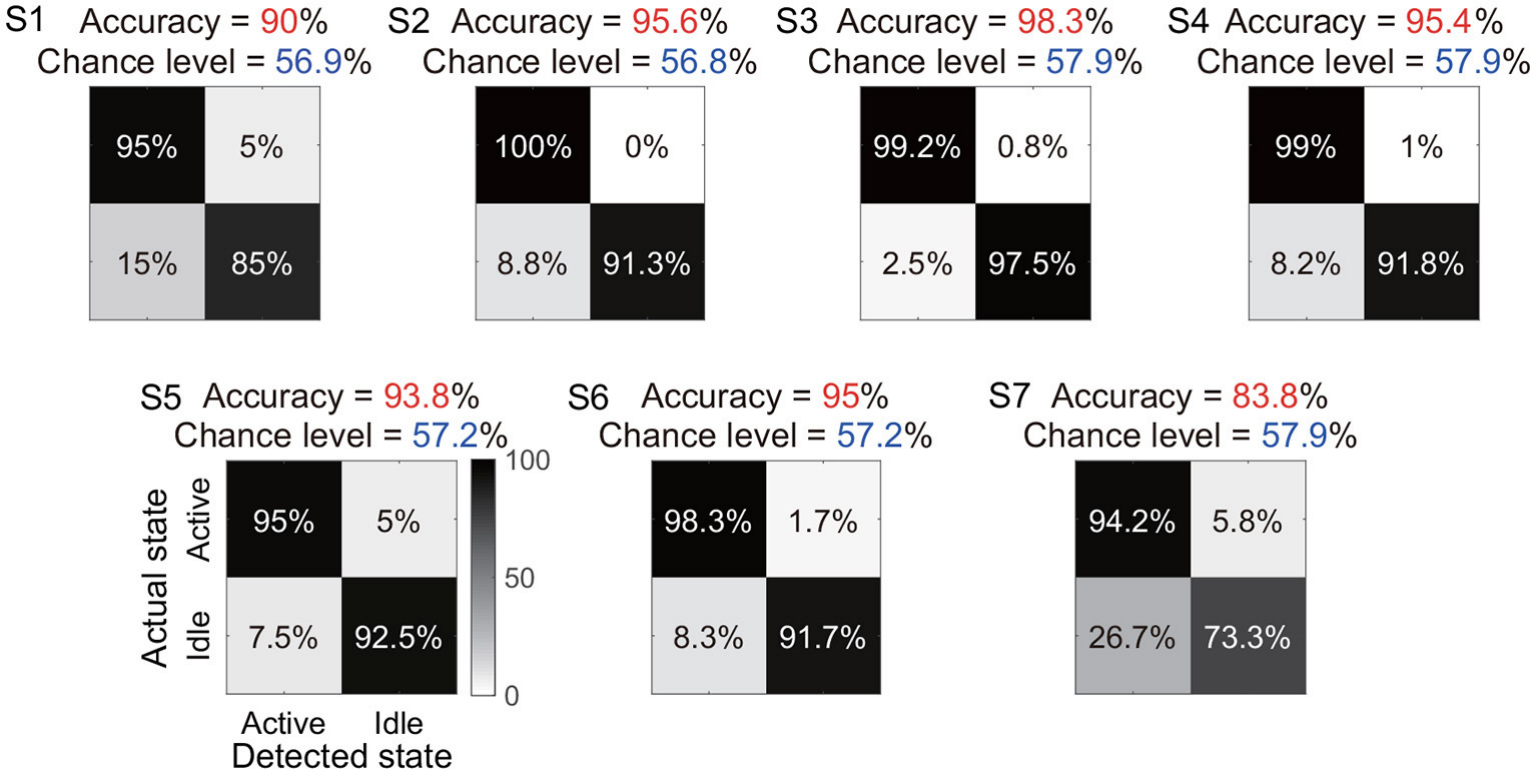
Total accuracy and confusion matrix for each subject. The total accuracy of all periods of active and idle states for each subject is marked with red, and the chance level is marked with dark blue. In the confusion matrix, each row represents a type of actual state, and each column represents a type of detected state.

Under the identified window length and the classifier, we further showed the total accuracy and confusion matrix for each subject (Figure 7). Six subjects (S1-S6) achieved a total accuracy above 90%, and the total accuracies of all subjects were significantly higher than their chance levels (*p* < 0.05). Subject S7 showed a relatively low total accuracy (83.8%) because 26.7% periods of actual idle state were identified as active state by the algorithm. In contrast, the accuracy for the actual active state was still at a high level of 94.2%.

### 3.2. Timing of the Onset and the End of Detected Active States

**Figure 8:**
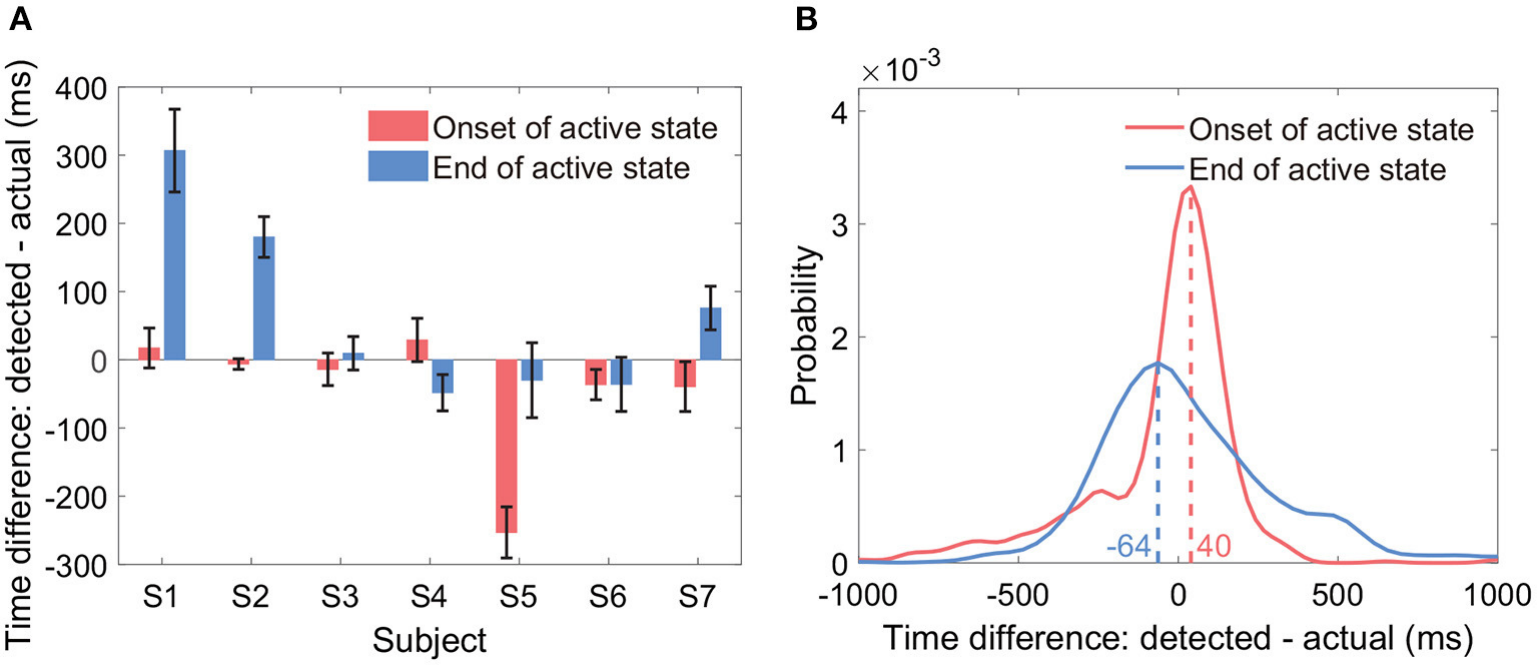
Timing of the onset and the end of detected active states. The sub-figures display the time difference between the detected active state onset and the actual active state onset, and the time difference between the detected active state end and the actual active state end. **(A)** Displays the two types of time differences within each subject (mean ± ste). **(B)** Displays the empirical distributions of the two types of time differences by group analysis of all active state periods from all subjects.

Results showed that the average onset time difference across all subjects was −43 ms, and the average end time difference was 65 ms (Figure 8A). By group analysis of all active state periods from all subjects, the empirical distributions of the two types of time difference peaked at −64 and 40 ms, respectively (Figure 8B). In general, the two types of time differences fluctuated around 0 ms across all active state periods. We displayed the time differences only if both the sensitivity and the precision were at a level of around 90%.

### 3.3. Evaluations for Different Features

**Figure 9:**
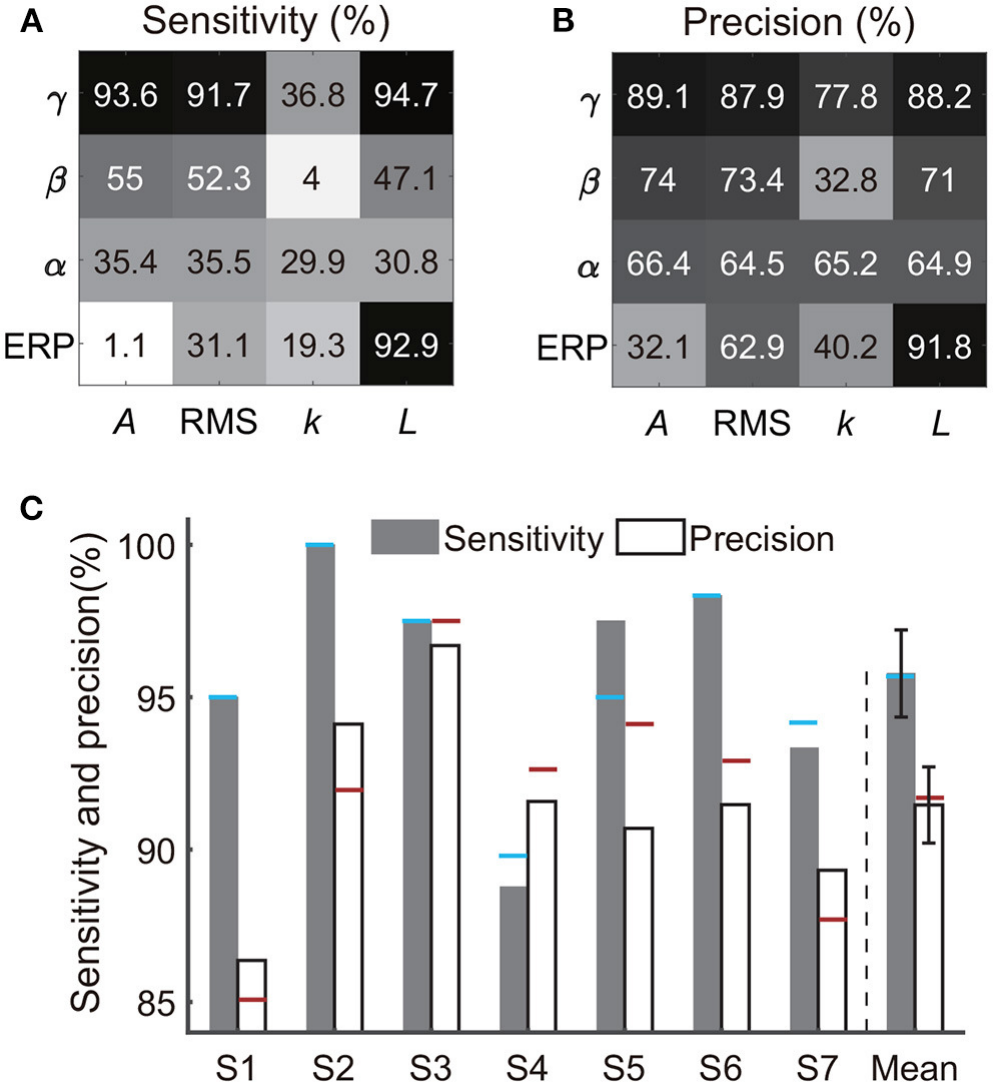
Performances of each single feature type and the four-type feature combination. Panels **(A,B)** exhibit sensitivities and precisions of 16 types of time-frequency or time-domain features separately, where each type of feature was a subset of the feature combination in Section 2.5 and Figure 4. The crossover point of the row and column indicates the result of the corresponding feature. Each row represents a type of signal trace, where γ, β, α, and ERP indicate the high-gamma power, beta power, alpha power, and the event-related potential, respectively. Each column represents a type of time-domain feature, where *A*, RMS, *k*, and *L* indicate the amplitude, root mean square, slope, and line-length, respectively. The value represents the mean accuracy across subjects. **(C)** Sensitivity and precision of the four-type feature combination. Error bars depict the standard error across subjects. The results were compared with the performances of the 16-type feature combination, where the blue horizontal line and the red horizontal line indicate the sensitivity and the precision calculated using the 16-type feature combination, respectively.

Besides the combination of time-frequency and time-domain features, we further evaluated the decoding performance of each single type of feature. Briefly, using each single type of feature adopted in Section 2.5 (e.g., line-length of high-gamma power trace, Figure 3), we separately implemented the classification again. Results from all subjects showed that the decoding accuracy varied across feature types (Figures 9A,B). For example, all features derived from alpha power resulted in low sensitivities (< 40%) and precisions (< 70%); all features derived from the slope of linear regression also resulted in low sensitivities (< 40%) and precisions (< 80%). Therefore, the following analysis rejected these invalid features. In contrast, high-gamma amplitude, high-gamma RMS, high-gamma line-length, and ERP line-length showed the highest sensitivities and precisions for all subjects. Within these four types of informative features, high-gamma line-length obtained the highest sensitivity (94.7%), and ERP line-length obtained the highest precision (91.8%). After redundant feature rejection, we recalculated the performance based on the combination of the above four types of features.

**Figure 10:**
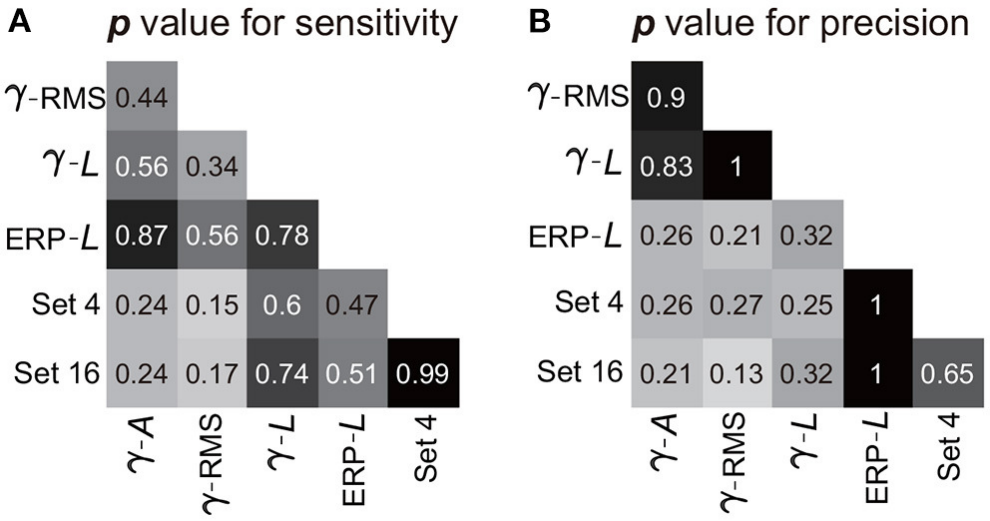
Statistic results of comparisons among features. Panels **(A,B)** exhibit the *p*-value of the Mann–Whitney *U*-test for the sensitivity and precision, respectively. Pairwise comparisons were performed among the features (or feature combination), where the crossover point of the row and column indicates the *p*-value between the corresponding two features (or feature combination). The symbols for features (γ, β, α, *A*, and *L*) are the same as those in Figure 9. Set 4 indicates the four-type feature combination, and Set 16 indicates the 16-type feature combination.

The combination of the four-type informative features achieved an average sensitivity of 95.8 ± 1.4% (mean ± ste) and an average precision of 91.5 ± 1.2% (Figure 9C). The highest sensitivity was in S2 (100%), and the highest precision was in S3 (96.7%). The lowest sensitivity was in subject S4 (88.8%), and the lowest precision was in S1 (86.4%). Importantly, Mann–Whitney *U*-tests suggested no significant difference between the performances calculated using the four-type feature combination and the performance calculated using all 16-type feature combination (Section 3.1), where the *p*-value for the sensitivity and the precision were 0.99 (Figure 10A) and 0.65 (Figure 10B), respectively, indicating that the four-type feature combination provided enough information. Meanwhile, though the sensitivity of the four-type feature combination was higher than the sensitivity of each single type of the feature, including the sensitivity of ERP line-length (92.9 ± 2.55%), these differences did not show statistical significance (*p* = 0.15–0.6, Mann–Whitney *U*-tests, Figure 10A). The differences between the precision of the four-type feature combination and the precision of the single type of informative feature did not show statistical significance, either (*p* = 0.25-1, Mann–Whitney *U*-tests, Figure 10B). Moreover, there was no significant difference among sensitivities of single types of features (*p* = 0.34-0.87, Figure 10A), and there was no significant difference among precisions of single types of features, either (*p* = 0.21-1, Figure 10B). Thus, these results suggested that each single type of informative feature encoded sufficient information. Though each single type of informative feature could detect the state transition independently in the current work, we still adopted the four-type feature combination in following analyses to reserve discriminant information maximally.

**Figure 11:**
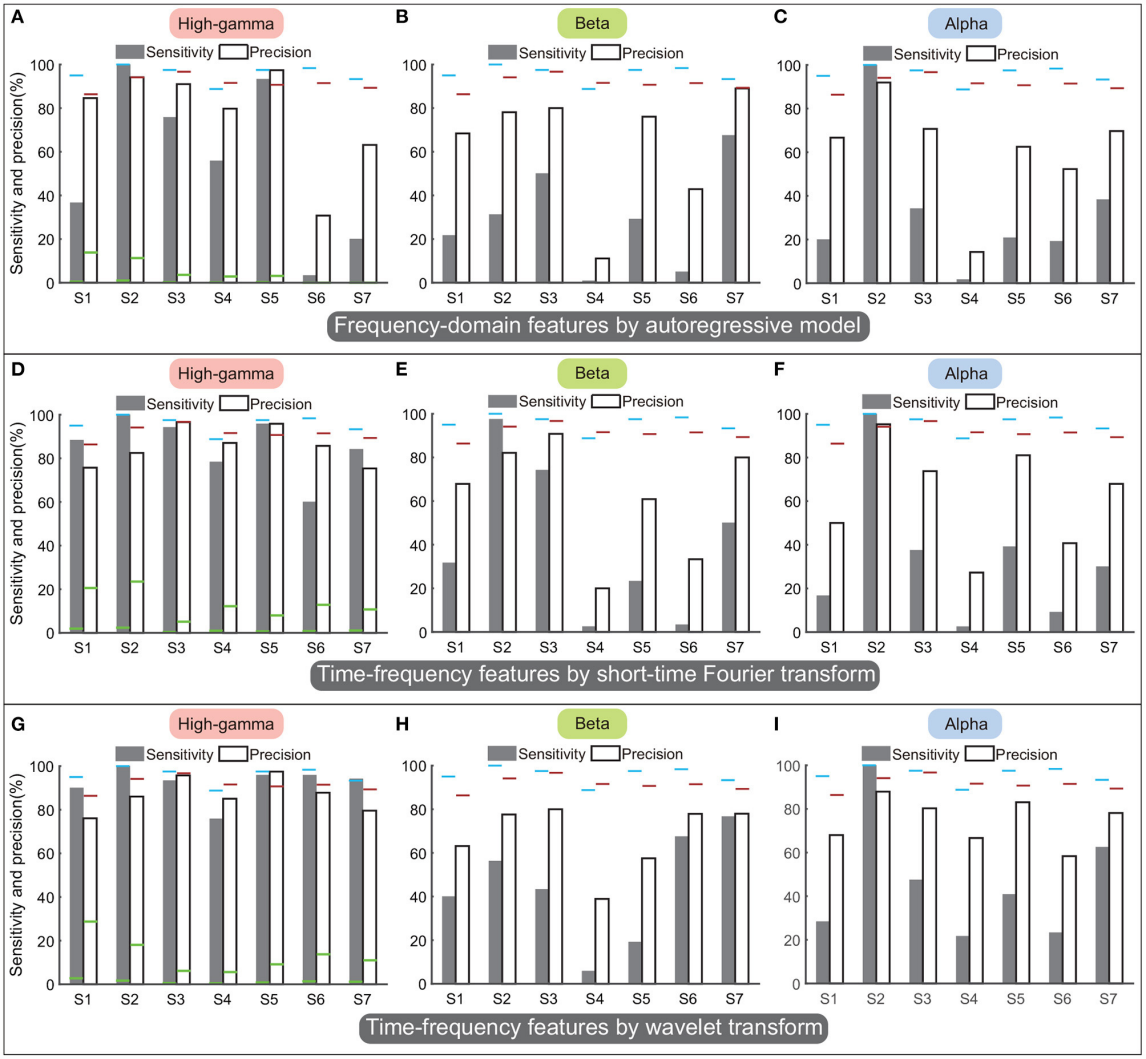
Performances of frequency-domain or time-frequency features calculated by autoregressive model, short-time Fourier transform, and wavelet transform. Panels **(A–C)** display the performances of autoregressive model. Panels **(D–F)** display the performances of short-time Fourier transform. Panels **(G–I)** display the performances of the wavelet transform. By each approach, the sensitivity and precision were calculated in the high-gamma, beta, and alpha bands separately. The results were compared with the performances of the above four-type feature combination (Figure 9C), where the blue horizontal line and the red horizontal line indicate the sensitivity and the precision calculated using the four-type feature combination, respectively. The green horizontal lines of each subject in **(A,D,G)** indicate the chance levels of the sensitivity and the precision corresponding to the features.

Figures 11A–C display performances of spectral amplitudes in the high-gamma, beta, and alpha bands calculated by the AR model. In general, these traditional frequency-domain features could not achieve satisfactory performance among all subjects. Though spectral amplitudes of the high-gamma band achieved the best performances among the three frequency bands, only subjects S2 and S5 obtained sensitivity and precision above 90%, and the sensitivity or precision was lower than 80% in other subjects (Figure 11A). The sensitivity and precision of subject S6 were especially low. Regarding the beta and alpha bands, the averaged sensitivity across subjects was only 36% and 35%. These frequency-domain features always failed to detect the state transition, and thus the classifier always resulted in sustained outputs of the same state. Therefore, the number of TP was limited in a small value but the number of FN could be large, accounting for low sensitivity and precision simultaneously (Figures 11B,C).

Figures 11D–F display performances of time-frequency features calculated by STFT, and Figures 11G–I display performances of time-frequency features calculated by wavelet transform. Results showed that time-frequency features in the high-gamma band (Figures 11D,G) outperformed features in the beta and alpha bands, similar to previous results. Thus we only compared the performances in the high-gamma band. The sensitivity and precision calculated by STFT were 85.8 ± 5.1% (mean ± ste) and 85.5 ± 3.2%, respectively (Figure 11D), and the sensitivity and precision calculated by wavelet transform were 92.1 ± 2.9% and 86.8 ± 3%, respectively (Figure 11G). By these two approaches, sensitivities and precisions for all subjects were higher than the chance levels significantly (*p* < 0.05). However, sensitivities and precisions of both wavelet transform and STFT were lower than the sensitivity and precision of our four-type feature combination (95.8 and 91.5%). Limited by the number of subjects, the comparisons could not show significant differences (*p* = 0.1–0.4, Mann–Whitney *U*-tests). For results of STFT, the sensitivity of subject S6 was below 60%; for results of the wavelet transform, sensitivity of S4 and precision of S1 were below 80%. In contrast, our four-type feature combination could always achieve remarkable sensitivities and precisions in these subjects, and even the minimum precision was up to 86.4% (S1). Overall, the four time-domain or time-frequency features we proposed have demonstrated their superiorities.

### 3.4. Performance of Three-Class Detection

**Figure 12:**
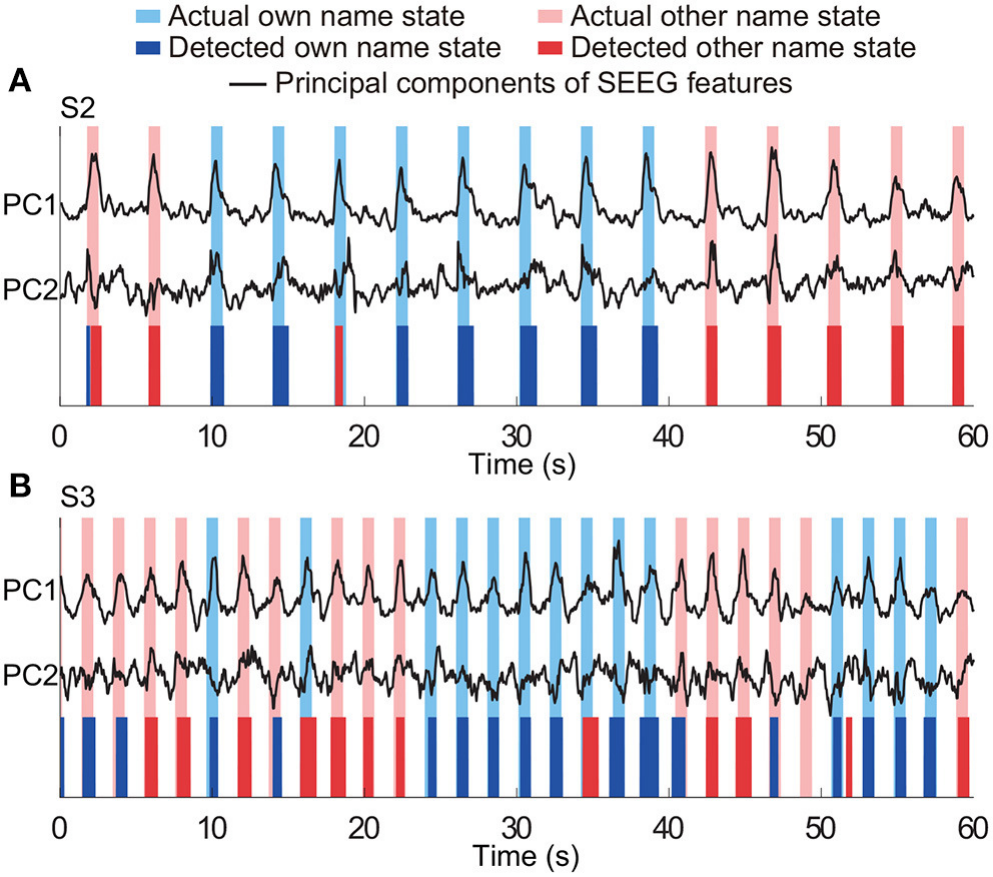
Representative results of three-class (own name, other name, and idle) state detection. Periods with white indicate the actual and detected idle states. Panels **(A,B)** are the results from two typical subjects (S2 and S3). The traces of principal components of SEEG features were normalized.

The three-class state detection was implemented using the above four-type optimal feature combination. Representative detection results of subjects S2 and S3 were shown in Figures 12A,B, respectively. In the example of S2, the first actual other name state period contained a very short period of detected own name state, and thus a false detection was counted. The third actual own name state period was misidentified as other name state. In the example of S3, there were more false detections between the own name state and other name state. Besides, there was an actual other name state period misidentified as idle state, and an actual idle state period misidentified as other name state. Averaged state detection accuracies across subjects can be seen in Table 3. The accuracies for own name state and other name state detection were 68.2 ± 5% and 70.8 ± 6.6%, respectively. About 29.8 ± 4.6% of actual own name state periods were misidentified as other name state periods, and 26.8 ± 6.6% of actual other name state periods were misidentified as own name state periods. In contrast, the accuracy for the idle state was still high (86.4 ± 2.7%).

**Table 3:** Averaged state detection accuracies across subjects (%).

**Actual/Detected**	**Idle**	**Own**	**Other**
Idle	**86.4** **±2.7**	5.9 ± 1.7	8.2 ± 2.4
Own	2 ± 1.3	**68.2** **±5**	29.8 ± 4.6
Other	2.4 ± 0.9	26.8 ± 6.6	**70.8** **±6.6**

*The values following the “±” indicate the standard error across subjects. The bold values indicate the accuracy of each class*.

## 4. Discussion

### 4.1. Influencing Factors of Detection Performance

The present study demonstrated the capability of using SEEG recordings to detect the active state against the idle state, and moreover, evaluated the influences of the detection configurations from multiple aspects, including the window length, the classifier, and features derived from ERP and different power traces. The current result showed that too long or too short windows could omit or conceal useful classification information. Under the current window settings, although performances of the different subjects did not show a united tendency as the window length changed, windows with a length of around 400 ms resulted in high and balanced sensitivity and precision in all subjects.

Our results suggested that HMM could produce the best performance among all the adopted classifiers under the current task. This may be because HMM models the matrix of state transition probability between samples (Blunsom, 2004; Elliott et al., 2008). In this situation, adjacent samples relate to each other, and the previous state provides references for estimation of the current state. Therefore, HMM is a popular statistical tool for modeling a wide range of time-series data. In contrast, other classifiers such as LDA, SVM, and RF, generally ignore the relationship between samples and deal with samples independently, even though these samples come from an ongoing data flow. Thus these three classifiers in our cases tended to generate independent and random outputs during the idle state, resulting in more false detections of the active state, and thus the precision was much lower.

### 4.2. Interpretations of Time-Domain Features and Frequency Band

The time-domain features in this study usually have specific implications, where the amplitude feature reflected the magnitude of the signal; the RMS feature could reflect the signal fluctuation (Pavlov et al., 2018), and the slope feature reflected the changing trend. Line-length is the running sum of the absolute differences between all consecutive samples within a predefined window, and thus the value of this feature will grow as the data sequence magnitude or signal variance increases (Koolen et al., 2014). Hence, line-length can be seen as an amplitude and frequency demodulator (Koolen et al., 2014). In this work, we extracted time-frequency features by performing the above time-domain operators to different band power traces or ERP of SEEG signals. The comparisons among decoding capabilities of various features were then implemented. Besides the high-gamma amplitude, the current study pioneered other three time-frequency or time-domain features derived from SEEG signals, including the high-gamma RMS, high-gamma line-length, and ERP line-length. These novel features could result in remarkable classification performance separately under the current task (Figures 9A,B), and thus provided effective guidance for SEEG studies. Some of the four informative features have been proven effective in ECoG or EEG studies using other paradigms. For example, the amplitude of high-gamma power extracted by the band-pass filter and Hilbert transform could distinguish the movement of each finger (Hotson et al., 2016). ERP line-length of ECoG could also decode different hand motions (Xie et al., 2015), and ERP line-length also worked in burst detection for EEG (Koolen et al., 2014). Meanwhile, the four-type feature combination's performance was higher than ones calculated by each single type of informative feature, although the *p*-values of comparisons were greater than 0.05, which might be caused by the limited number of subjects. Further, the performance of the four-type feature combination was at the same level as the performance achieved using all features (Figure 9C), which was beneficial to the decoding from the view of computational efficiency.

The excellent performances of the high-gamma band reported in this study had potential physiological interpretations. The high-gamma band is considered to reflect the local neural population's activity directly around or underneath the recording electrode (Ray et al., 2008). This means that the high-gamma activity indicates the excitability of the local region (Mukamel et al., 2005; Cardin et al., 2009; Miller et al., 2014; Parvizi and Kastner, 2018). Therefore, high-gamma activities may, to a large extent, reflect detailed neural information. Moreover, the superiority of high-gamma band reported in this work is also in agreement with previous BCI studies, such as spoken sentence decoding (Anumanchipalli et al., 2019; Moses et al., 2019), hand gestures (Chestek et al., 2013; Branco et al., 2017) and upper limb joints prediction (Thomas et al., 2019). Additionally, Miller et al. (2016) predicted the onset and type of visual stimulus from continuous data using broadband activities. The broadband activity extracted from the field potential has been shown to correlate with neuronal firing rate (Miller et al., 2009, 2016), and thus achieved superior detection performance, similar to findings in our study. In the current study, features derived from alpha and beta bands showed relatively poor performances. One possible explanation might be that features derived from slow-frequency oscillations in field potential had a relatively long variation period, and thus these features could not capture the rapidly changing external stimuli. Similarly, the beta band always showed poorer performance than the high-gamma band in tasks of hand gesture decoding (Chestek et al., 2013). Unlike the high-gamma band, low-frequency oscillations are considered as carrier frequencies for communication between distant brain regions (Brovelli et al., 2004; Knyazev et al., 2011; Potes et al., 2014; Parvizi and Kastner, 2018). Therefore, intracranial low-frequency oscillations were more commonly used to explore functional connectivity among different brain regions (Kirkby et al., 2018; Goodale et al., 2019). Regarding the ERP, its line-length also showed excellent performance. ERP contains both exogenous components and endogenous components. The exogenous components are modulated by physical attributes of stimuli but not by cognitive processes (Coles and Rugg, 1995; Cygan et al., 2014), whereas the endogenous components are considered related to cognitive processes, reflecting decision making, stimulus evaluation, and recognition (Coles and Rugg, 1995; Smigielski et al., 2020). During the ongoing process of name presentation in this study, the processing of physical attributes of auditory stimuli coexisted with cognitive processes constantly. Therefore, the ERP time series might be the integration of exogenous potentials and endogenous potentials. Abundant inner biological mechanisms of ERP might be the reason for its remarkable tracking of the rapidly changing external stimuli.

### 4.3. Implications

The processing of the acoustic name stimulus in the human brain involved a distributed connectivity network (Northoff and Bermpohl, 2004; Davey et al., 2016). For example, the acoustic name stimulus activates not only low-level auditory sense in the primary auditory cortex (mainly in the transverse temporal gyrus and the superior temporal gyrus) (Pickles, 2013; Nakai et al., 2017), but also activates high-level cognition in other structures such as the medial prefrontal cortex, inferior parietal lobule (Davey et al., 2016), the insula (Qin et al., 2012; Ye et al., 2021), and the fusiform (Carmody and Lewis, 2006). Parts of these findings by fMRI and scalp EEG measurements could be verified in the current study, showing that activated brain regions for the acoustic name stimulus distributed broadly in the temporal lobe, parietal lobe, and deeper structures such as the insula and fusiform (Table 2). Hence, all subjects showed noteworthy detection performance for the active state despite individual differences in electrode implantation. These facts might suggest that comprehensive utilization of neural responses from multiple distributed regions could improve the decoding performance and hence are recommended for further BCI applications. This idea has been implemented in previous SEEG-based motor decoding studies, which investigated the decoding performance of different regions and their combination. For example, while the primary motor cortex is always known as the optimal region to decode hand and foot movements and forces (Vadera et al., 2013; Murphy et al., 2016), other regions such as the primary somatosensory cortex and the posterior parietal cortex still provide supplementary BCI control signals (Branco et al., 2017; Wang et al., 2020).

As discussed in Section 4.1, compared with other classifiers, the GMM-HMM scheme has been proved suitable for spontaneous state detection owing to its modeling for the state transition (see Section 2.6). The decoding capability of the GMM-HMM scheme deserves further investigation using other BCI paradigms besides the auditory experimental paradigm in this study.

### 4.4. Limitations and Future Work

Even though we have demonstrated the availability of various SEEG features for active state detection, results showed that it was relatively hard to distinguish the two stimuli from the continuous signals under the current detection strategy, which might be caused by several possible reasons. First, we might not have enough sampling points within the critical regions that show selective responses because of the limited number of subjects. Second, in the current classification scheme, each feature only made a general description for a sliding window instead of several values at different time points within the window, which might not capture subtle differences between the two stimuli. In future work, we would adopt a cascade classification scheme to overcome this problem, where the HMM detects the active state onset first, and then another classifier could distinguish the type of the stimulus using the information at different time points within the active state (Ye et al., 2021). Based on a sufficient number of subjects in the future, we would reopen the investigation on statistical significance of comparisons between the four-type feature combination and each single type of informative feature. Additionally, during the classification process, this work used all the most informative channels together, however, as is well-known, different brain regions play distinguished roles during the task. Therefore, evaluating the contribution of single regions to the decoding separately is still essential and needs to be addressed using a larger number of subjects in the future.

## 5. Conclusion

Distributed SEEG recordings provide a new perspective to investigations on the link between neural activities in the human brain and external auditory stimuli. In this study, we proposed a GMM-HMM framework for auditory active state detection using continuous SEEG signals, by which the onset and the end of auditory stimuli were estimated. We have verified the effectiveness of the single type of time-frequency or time-domain features, and the combination of these features has also been proven effective in spontaneous state detection. As a preliminary investigation, the findings of this study provided important clues for further SEEG-based BCI studies.

## Data Availability Statement

The raw data supporting the conclusions of this article are available from the corresponding author upon reasonable request.

## Ethics Statement

The studies involving human participants were reviewed and approved by Ethics Committee of Huashan Hospital, Shanghai, China. The patients/participants provided their written informed consent to participate in this study.

## Author Contributions

HY, ZF, and LC: conceptualization. HY: methodology. HY and GL: software. HY and ZF: formal analysis, writing—original draft, and visualization. HY, ZF, XS, XZ, and LC: investigation. JH, XS, XZ, and LC: resources. HY, ZF, and ZW: data curation. GL and ZW: writing—review and editing. XZ, LC, and JH: supervision. XZ, LC, and XS: project administration and funding acquisition. All authors read and approved the final version of the manuscript.

## Funding

This work was supported in part by the China National Key R&D Program (Grant No. 2018YFB1307200) and the National Natural Science Foundation of China (Grant No. 91948302).

## Conflict of Interest

The authors declare that the research was conducted in the absence of any commercial or financial relationships that could be construed as a potential conflict of interest.

## Publisher's Note

All claims expressed in this article are solely those of the authors and do not necessarily represent those of their affiliated organizations, or those of the publisher, the editors and the reviewers. Any product that may be evaluated in this article, or claim that may be made by its manufacturer, is not guaranteed or endorsed by the publisher.
